# The Effectiveness and Safety of Commonly Used Injectates for Ultrasound-Guided Hydrodissection Treatment of Peripheral Nerve Entrapment Syndromes: A Systematic Review

**DOI:** 10.3389/fphar.2020.621150

**Published:** 2021-03-05

**Authors:** Montana Buntragulpoontawee, Ke-Vin Chang, Timporn Vitoonpong, Sineenard Pornjaksawan, Kittipong Kitisak, Surasak Saokaew, Sukrit Kanchanasurakit

**Affiliations:** ^1^ Department of Rehabilitation Medicine, Faculty of Medicine, Chiang Mai University, Chiang Mai, Thailand; ^2^ Department of Physical Medicine and Rehabilitation, National Taiwan University Hospital Bei-Hu Branch, Taipei, Taiwan; ^3^ Department of Rehabilitation Medicine, Faculty of Medicine, Chulalongkorn University, Bangkok, Thailand; ^4^ Rehabilitation Clinic, Bangkok Hospital Chiang Mai, Chiang Mai, Thailand; ^5^ Division of Pharmacy Practice, Department of Pharmaceutical Care, School of Pharmaceutical Sciences, University of Phayao, Phayao, Thailand; ^6^ Center of Health Outcomes Research and Therapeutic Safety (Cohorts), School of Pharmaceutical Sciences, University of Phayao, Phayao, Thailand; ^7^ Unit of Excellence on Clinical Outcomes Research and IntegratioN (UNICORN), School of Pharmaceutical Sciences, University of Phayao, Phayao, Thailand; ^8^ Division of Pharmaceutical Care, Department of Pharmacy, Phrae Hospital, Phrae, Thailand

**Keywords:** entrapment neuropathy, ultrasound-guided hydrodissection, peripheral nerve, perineural injection, injectate, carpal tunnel syndrome, cubital tunnel syndrome

## Abstract

**Background:** Peripheral nerve entrapment syndromes commonly result in pain, discomfort, and ensuing sensory and motor impairment. Many conservative measures have been proposed as treatment, local injection being one of those measures. Now with high-resolution ultrasound, anatomical details can be visualized allowing diagnosis and more accurate injection treatment. Ultrasound-guided injection technique using a range of injectates to mechanically release and decompress the entrapped nerves has therefore developed called hydrodissection or perineural injection therapy. Several different injectates from normal saline, local anesthetics, corticosteroids, 5% dextrose in water (D5W), and platelet-rich plasma (PRP) are available and present clinical challenges when selecting agents regarding effectiveness and safety.

**Aims:** To systematically search and summarize the clinical evidence and mechanism of different commonly used injectates for ultrasound-guided hydrodissection entrapment neuropathy treatment.

**Methods:** Four databases, including PubMed, EMBASE, Scopus, and Cochrane were systematically searched from the inception of the database up to August 22, 2020. Studies evaluating the effectiveness and safety of different commonly used injectates for ultrasound-guided hydrodissection entrapment neuropathy treatment were included. Injectate efficacy presents clinical effects on pain intensity, clinical symptoms/function, and physical performance, electrodiagnostic findings, and nerve cross-sectional areas. Safety outcomes and mechanism of action of each injectate were also described.

**Results:** From ten ultrasound-guided hydrodissection studies, nine studies were conducted in carpal tunnel syndrome and one study was performed in ulnar neuropathy at the elbow. All studies compared different interventions with different comparisons. Injectates included normal saline, D5W, corticosteroids, local anesthetics, hyaluronidase, and PRP. Five studies investigated PRP or PRP plus splinting comparisons. Both D5W and PRP showed a consistently favorable outcome than those in the control group or corticosteroids. The improved outcomes were also observed in comparison groups using injections with normal saline, local anesthetics, or corticosteroids, or splinting. No serious adverse events were reported. Local steroid injection side effects were reported in only one study.

**Conclusion:** Ultrasound-guided hydrodissection is a safe and effective treatment for peripheral nerve entrapment. Injectate selection should be considered based on the injectate mechanism, effectiveness, and safety profile.

## Introduction

Peripheral nerves are susceptible to pressure-induced injury as they travel along different anatomical structures resulting in entrapment neuropathy (Trescot and Brown, 2015). Pressure-induced injury can result from mechanical compression, constriction, overstretching, or edema. The cause of compression can be exogeneous; caused by instruments or other non-bodily structures, or endogeneous; caused from the patient’s body (Toussaint et al., 2010). In cases of endogenous causes, the compression can be external to the nerve or internal, as the compressive structure originates from one of the nerve’s components itself. Entrapment may occur at various sites in the body whether between muscles or bones, around blood vessels, across joints, and through tunnels or fascial penetration sites (Toussaint et al., 2010). The most common site of entrapment is the median nerve at the wrist or carpal tunnel syndrome (CTS) and the second most common is the ulnar nerve at the elbow or cubital tunnel syndrome (CuTS) (Doughty and Bowley, 2019). Other reported less common sites include lateral femoral cutaneous nerve, lateral antebrachial cutaneous nerve, and medial superior cluneal nerves (Tagliafico et al., 2011; Chang et al., 2017; Wu and Boudier-Revéret, 2019). Entrapment can disturb sensory and/or motor function resulting in neuropathic pain, discomfort, and weakness (Toussaint et al., 2010; Schmid et al., 2013). Nerve compression leads to segmental intraneural ischemia disrupting the blood-nerve barrier and dysfunction of the intraneural circulation, intraneural edema formation, and ectopic impulse generation of both mechanosensitive and nociceptive neurons resulting in neuropathic pain with varying severity (Schmid et al., 2013; Trescot and Brown, 2015). Activated C-fibers may produce and release pain-producing and degenerative neuropeptides such as substance P and calcitonin gene-related peptide (CGRP) resulting in chronic neurogenic inflammation (Ji et al., 2018). With prolonged compression, demyelination and axonal loss follow, as well as nerve fascicles swelling leading to epineural fibrosis. Many treatment options are available to counter the effect of entrapment, conservative measures include splinting, tendon and nerve gliding exercise, physical modalities, and corticosteroids injection (Huisstede et al., 2010; Kooner et al., 2019). Patients who respond poorly to those measures become candidates for surgical decompression or reconstruction (Lauder et al., 2019). At present, high-resolution ultrasound plays important role in the diagnosis of entrapment neuropathy and guided injection delivering a range of injectates, for example, normal saline, corticosteroids, local anesthetics, dextrose, and platelet-rich plasma (PRP). This procedure, known as hydrodissection or perineural injection, provides not only a mechanical effect to release and decompress the entrapped nerves but also a pharmacological effect relieving pain and promoting recovery through numerous mechanisms (Chang et al., 2020; Lam et al., 2020; Reeves and Rabago, 2020). There has been a considerable increase in interest and publications of this procedure regarding the benefits and disadvantages or adverse effects of each different agent (Catapano et al., 2020; Lam et al., 2020; Lin et al., 2020). As clinicians planning to perform such a procedure, agent selection is usually based on effectiveness and safety. Therefore, the present systematic review aims to present the effectiveness and safety of different commonly used injectates for ultrasound-guided hydrodissection entrapment neuropathy treatment, explain relevant mechanism of action and discuss practical issues with agent selection as well as highlight knowledge gaps found.

## Methods

This systematic review was carried out and reported following the Preferred Reporting Items for Systematic Reviews and Meta-Analyses (PRISMA) statement (Moher et al., 2009).

### Data Sources and Search Strategy

EMBASE, Scopus, Cochrane, and PubMed were systematically searched from their establishment to August 22, 2020. The Medical Subject Headings (MeSH) were utilized as applicable. The bibliography lists of associated articles were explored. Strategic search terms included “nerve hydrodissection”, “injectates”, “steroid”, “saline”, “platelet-rich plasma”, and “5% dextrose” with slight modifications based on the database. There was no language restriction.

### Study Selection

From these articles, the included studies were selected according to the following criteria: 1) carried out in patients age over 18 years with peripheral nerve entrapment syndrome; 2) patients received guided ultrasound; and 3) clinical effects of intervention were evaluated comparing perineural injections with non-surgical treatments for peripheral nerve entrapment syndrome. Animal studies and studies are not displayed as original research such as comments, expert opinions, case reports, case series, conference meeting abstracts, surveys, reviews, editorials, systematic reviews, meta-analyses, observational study, and letters were excluded. Two investigators (M.B. and S.K.) separately assessed each title, abstract, and full-text article for possibly eligible studies. Disagreements were resolved by consensus.

### Data Extraction and Outcome Measures

Data extractions from all possibly appropriate articles were performed independently by the two reviewers (M.B. and S.K.). When discrepancies occurred, they were resolved by consensus discussions with a third reviewer (S.S.). The data extracted and described included the following: region, study design, diagnosis, treatment allocation, characteristics of participants (such as age, sex, and the number of participants), follow-up interval, efficacy outcome, and safety outcome. The outcomes of interest were pain, measured by visual analog scale (VAS), clinical symptoms and function measured by the Boston Carpal Tunnel Questionnaire (BCTQ) separately as a symptom severity scale (BCTQs) and a functional status scale (BCTQf) or as a single combined scale (BCTQ combined), and lastly, by the Quick-Disability of Arm Shoulder and Hand (Q-DASH) questionnaire. Also used were participant-rated clinical outcome assessments by subjective symptom changes and global assessment of treatment results, other physical performances were measured by finger pinch strength (kg), monofilament testing score, static and dynamic two-point discrimination scores. Electrodiagnostic findings (EDS) were measured by sensory nerve conduction velocity (SNCV, m/s), distal motor latency (DML, ms), motor nerve conduction study (MNCS, m/s), distal compound motor action potential amplitude (CMAP, mV), sensory latency (ms), and sensory nerve action potential amplitude (SNAP, mV), and ultrasound measurement of nerve cross-sectional area (CSA).

### Quality Assessment

The quality of the individual study was appraised independently by two investigators (S.K. and S.S.) using the Cochrane Risk-of-bias tool 2.0 (RoB 2.0) for randomized controlled trials. This tool includes six domains for methodological evaluation: 1) random sequence generation, 2) allocation concealment, 3) blinding of participants and personnel, 4) blinding of outcome assessment, 5) incomplete outcome data, and 6) selective reporting. Each study was classified as having a low risk, high risk, or unclear risk. Disagreements were resolved by discussion.

### Statistical Analysis

Overall effects were analyzed and stratified according to clinical effect and intervention for treating peripheral nerve entrapment syndrome. If data was available, a pairwise or network meta-analysis with a DerSimonian-Laird random-effects model was used to estimate treatment effects, pooled risk ratios (RR), or weighted mean differences (WMD) along with 95% confidence intervals (CI) for dichotomous and continuous outcomes, respectively. Statistical heterogeneity between studies was assessed using the *I* ^2^ values. *I* ^2^ values lower around 25%, 25%–75%, and greater than 75% indicate low, moderate, and high heterogeneity, respectively (DerSimonian and Laird, 1986; Higgins et al., 2003). The software used for data analysis was STATA version 14 (STATA Corp, College Station, TX, USA).

## Results

### Study Selection

**FIGURE 1:**
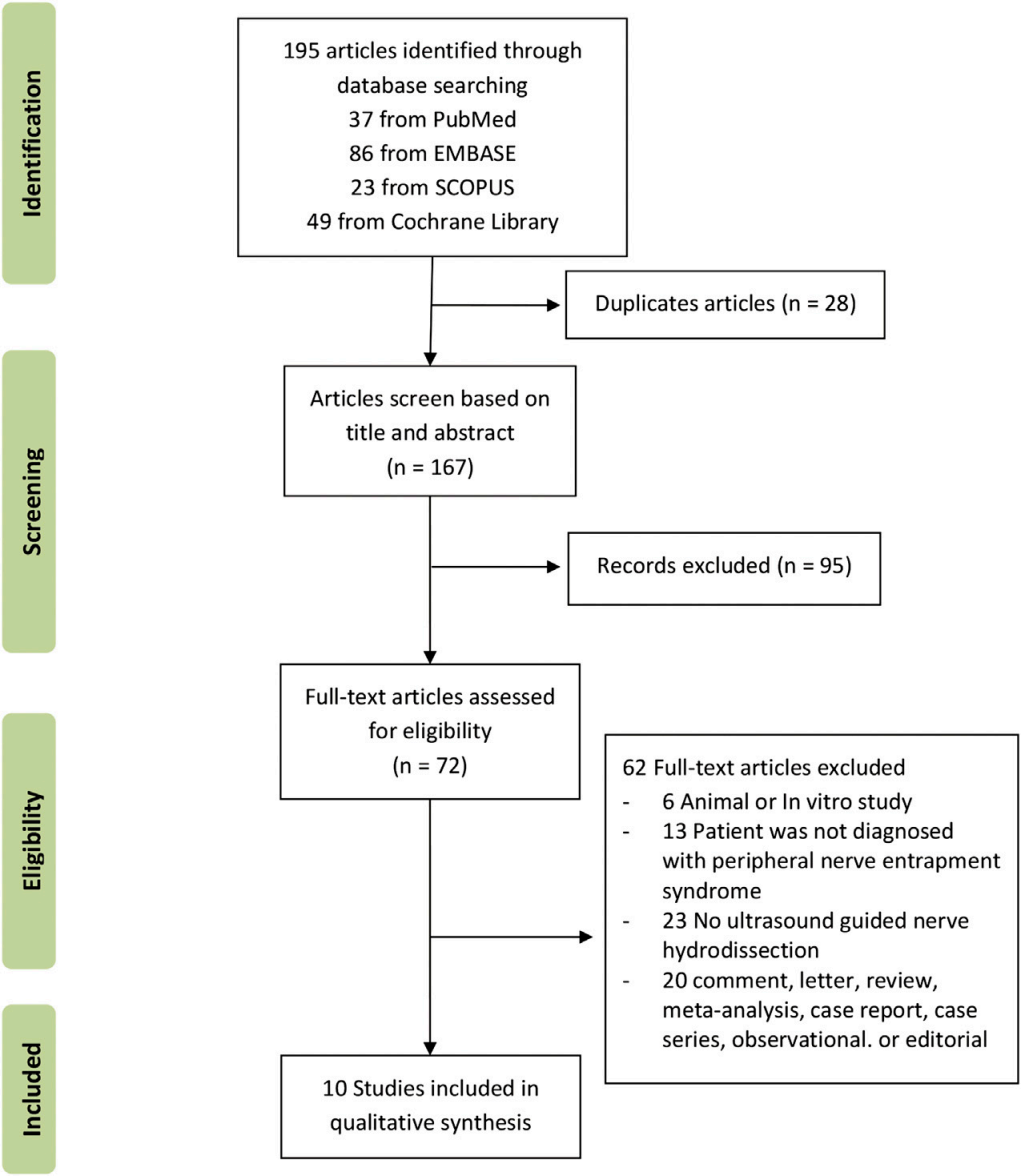
PRISMA flow diagram summary of the study selection process.

A total of 195 records were identified through database searching (n = 195). A total of 167 records remained after duplicates were removed. Of the remaining 167 records, ninety-five were deemed ineligible based on title and abstract. Of the 72 articles qualified for a full-text review, sixty-two full-text articles were excluded because they did not meet the study eligibility criteria. The flow chart in Figure 1 presents the results describing the exclusions at different stages during the review process. Ten studies were included in this systematic review (van Veen et al., 2015; Wu et al., 2017a; Wu et al., 2017b; Malahias et al., 2018; Roghani et al., 2018; Wu et al., 2018; Alsaeid, 2019; Güven et al., 2019; Senna et al., 2019; Shen et al., 2019).

### Characteristics of Included Studies

The general characteristics of the included studies are presented in Table 1. Of the ten included studies, seven studies were conducted in patients with mild to moderate carpal tunnel syndrome (CTS), two in patients with moderate carpal tunnel syndrome (CTS), and one in patients with cubital tunnel syndrome (CuTS). Four studies were from Taiwan, two were from Egypt, one was from Turkey, one was from Greece, one was from the United States and one was from the Netherlands. The study design of the ten studies included five randomized double-blind controlled trials, three randomized single-blind controlled trials, one triple-blind randomized controlled trial, and one prospective quasi-experimental trial. This systematic review included 569 patients with 570 affected wrists. The majority (>75%) of the overall participants were women in eight out of ten studies. The average age in the patient group in the included studies ranged from 38.3 to 66.1 years.

**TABLE 1:** Characteristics of the included randomized controlled studies using different injectates (corticosteroids, dextrose, PRP) for ultrasound-guided injection treatment of peripheral nerve entrapment.

Author, year, region	Study design	Diagnosis	Treatment allocation	Participant characteristics			Follow-up interval (months)	Efficacy outcome	Safety outcome
			Intervention: Comparison	Number of participants (wrists)	Mean age (years)	Female (%)			
VanVeen et al., 2015, Netherlands	Randomized double blind-controlled trial	UNE by clinical EDS or US	Methylprednisolone	30 (30)	56 ± 15	40	3	***Participate-rated Symptom change and severity as sensory, neuro exam,*** EDS, CSA of UN	1 placebo patient-reported pain at injection site at injection site,
			Normal saline	25 (25)	53 ± 12	64			
Wu et al., 2017a, Taiwan	Randomized single-blind controlled trial	Mild to moderate CTS by clinical + EDS	PRP	30 (30)	57.9 ± 1.5	90	1,3,6	***VAS,*** BCTQ, CSA of MN, EDS, finger pinch strength	No side effects or nerve trauma observed
			Splint	30 (30)	54.3 ± 1.3	83.3			
Wu et al., 2017b, Taiwan	Randomized double-blind controlled trial	Mild to moderate CTS by clinical + EDS	D5W	25 (30)	58.5 ± 2.3	86.7	1,3,6	***VAS,*** BCTQ, CSA of MN, EDS, global assessment of treatment results	No adverse effects, complications or nerve trauma observed
			Normal saline	24 (30)	58.1 + 1.9	80.0			
Malahias, et al., 2018, Greece	Randomized double-blind controlled trial	Mild to moderate CTS by clinical diagnosis	PRP	26 (26)	60.4 ± 14.3	NA	1,3	***VAS,*** Q-DASH, Delta-CSA of MN	No complication
			Normal saline	24 (24)	57.1 ± 16.1				
Roghani et al., 2018, USA	A triple-blind randomized controlled trial	Moderate CTS by clinical + EDS, age	Group I: 80 mg triamcinolone	32 (32)	66.1 ± 13.4	68.6	0.5,3,6	VAS, BCTQ, CSA of MN, EDS	NA
		> 50 years	Group II: 40 mg triamcinolone	32 (32)	66.1 ± 1.0	87.5			
			Group III: lidocaine	30 (30)	63.4 ± 10.7	90			
Wu et al., 2018, Taiwan	Randomized double-blind clinical trial	Mild to moderate CTS by clinical + EDS	D5W	27 (27)	58.6 ± 2.2	81.4	1,3,4,6	***VAS,*** BCTQ, EDs, CSA of MN, global assessment of treatment results	No side effects or complications
			Triamcinolone	27 (27)	54.3 ± 2.0	77.7			
Alsaeid, 2019, Egypt	Randomized double-blinded controlled trial	Mild to moderate CTS by clinical + EDS + US	Hyaloronidase	20 (20)	40.2 ± 10.5	55	0.25, 1,3,6	BCTQ, EDS, CSA of MN	No allergy from hyaluronidase
			Dexamethasone	20 (20	42.8 ± 8.3	50			
Güven et al., 2019, Turkey	Prospective quasiexperimental	Mild to moderate CTS by clinical + EDS	PRP + splinting	18 (20)	47.5	94.4	1	BCTQ, EDS, CSA of MN, monofilament testing, static and dynamic 2PD testing score	No complication
				12 (20)	50	91.6			
Senna et al., 2019, Egypt	Randomized single-blinded controlled trial	Mild to moderate CTS by clinical + EDS + US	PRP	43 (43)	38.3 ± 6.4	81.4	1,3	***VAS,Paresthesia, Phalen’s,Tinel’s,***BCTQ, EDS, CSA of MN	No recorded side effects
			Methylprednisolone	42 (42)	40.7 ± 9.4	85.7			
Shen et al., 2019, Taiwan	Randomized single-blind trial	Moderate CTS by clinical + EDS	PRP	26 (26) 26 (26)	56.8 ± 10.7	96.284.6	1,3,6	***BCTQ,*** EDS, CSA of MN	No serious adverse effects
			D5W		58.5 ± 11.7				

CTS: carpal tunnel syndrome, UNE: ulnar neuropathy at the elbow, VAS: visual analogue scale, EDS: electrodiagnostic study, US: ultrasound study, CSA: cross-sectional area, UN: ulnar nerve, D5W: 5% dextrose in water, MN: median nerve, BCTQ: Boston carpal tunnel syndrome questionnaire, NA: Not available, Q-DASH: Quick Disabilities of Arm, Shoulder, and Hand questionnaire, Delta-CSA: cross-sectional area difference of the median nerve’s surface at the tunnel’s inlet, minus the median nerve’s surface proximal to the tunnel and overpronator quadratus. 2PD: two-point discrimination, 0.5 months represents 2 weeks duration, 0.25 months represents 1 week duration. REMARK: Data present as mean ± standard deviation, * presented as mean ± standard error. Primary efficacy outcomes are bold and italicized.

All ten studies compared the different ultrasound-guided interventions to different comparison injectate or other conservative treatment methods, none compared a matched intervention and comparison group. Intervention injectate ranges from corticosteroids, 5% dextrose (D5W), platelet-rich plasma alone, or platelet-rich plasma (PRP) combined with splinting as an intervention and hyaluronidase. Three studies used normal saline (NSS) as a control injectate, each study compared corticosteroids, D5W, and PRP, respectively, as an intervention to NSS control (van Veen et al., 2015; Wu et al., 2017b; Malahias et al., 2018). Two studies used splinting as a control conservative treatment method, each study compared PRP and PRP combined with splinting, respectively, as an intervention to splinting control (Wu et al., 2017a; Güven et al., 2019). The remaining five studies compared two different injectates or different doses of an injectate as the following details, one study compared D5W with corticosteroids, one study compared different doses of corticosteroids with local anesthetics, one study compared hyaluronidase with corticosteroids as an adjuvant to local anesthetics (LA), one study compared PRP with steroid and one study compared PRP with D5W (Roghani et al., 2018; Wu et al., 2018; Alsaeid, 2019; Senna et al., 2019; Shen et al., 2019). Regarding efficacy outcome measurement used, the visual analog scale for pain (VAS) was used in six studies (Wu et al., 2017a; Wu et al., 2017b; Malahias et al., 2018; Roghani et al., 2018; Wu et al., 2018; Senna et al., 2019). Clinical symptoms and function measured by the Boston carpal tunnel questionnaire (BCTQ) separately as a symptom severity scale (BCTQs) and functional status scale (BCTQf) were used in seven studies (Wu et al., 2017a; Wu et al., 2017b; Wu et al., 2018; Alsaeid, 2019; Güven et al., 2019; Senna et al., 2019; Shen et al., 2019). BCTQ was used as a combined single scale in one study (Roghani et al., 2018). Participant-rated clinical outcome assessment by subjective symptom change was used in one study (van Veen et al., 2015) and two studies by the same investigator used a global assessment of treatment results as a participant-rated tool (Wu et al., 2017b; Wu et al., 2018). For physical performance, one study measured finger pinch strength (Wu et al., 2017a), one study measured monofilament testing scores, static and dynamic two-point discrimination scores (Güven et al., 2019). Nine studies measured electrodiagnostic parameters (van Veen et al., 2015; Wu et al., 2017a; Wu et al., 2017b; Roghani et al., 2018; Wu et al., 2018; Alsaeid, 2019; Güven et al., 2019; Senna et al., 2019; Shen et al., 2019). All ten studies measured the cross-sectional area of the investigated nerve (CSA) (van Veen et al., 2015; Wu et al., 2017a; Wu et al., 2017b; Malahias et al., 2018; Roghani et al., 2018; Wu et al., 2018; Alsaeid, 2019; Güven et al., 2019; Senna et al., 2019; Shen et al., 2019). VAS was the primary outcome in six studies (Wu et al., 2017a; Wu et al., 2017b; Malahias et al., 2018; Roghani et al., 2018; Wu et al., 2018; Senna et al., 2019), while BTCQ was the primary outcome in one study (Shen et al., 2019). All ten studies used an in-plane ultrasound-guided injection technique (van Veen et al., 2015; Wu et al., 2017a; Wu et al., 2017b; Malahias et al., 2018; Roghani et al., 2018; Wu et al., 2018; Alsaeid, 2019; Güven et al., 2019; Senna et al., 2019; Shen et al., 2019). The shortest duration for post-injection follow up was at one week (0.25 months) interval in one study (Alsaeid, 2019), the maximum follow-up duration was six months in six studies (Wu et al., 2017a; Wu et al., 2017b; Roghani et al., 2018; Wu et al., 2018; Alsaeid, 2019; Shen et al., 2019). Nine studies reported side effects or adverse events outcomes (van Veen et al., 2015; Wu et al., 2017a; Wu et al., 2017b; Malahias et al., 2018; Wu et al., 2018; Alsaeid, 2019; Güven et al., 2019; Senna et al., 2019; Shen et al., 2019). Only one of those nine studies reported adverse events after injection while the other eight studies reported no post-injection side effects or adverse events (van Veen et al., 2015). One study, however, did not mention these side effects or adverse events outcomes (Roghani et al., 2018).

### Assessment of Risk of Bias

**FIGURE 2:**
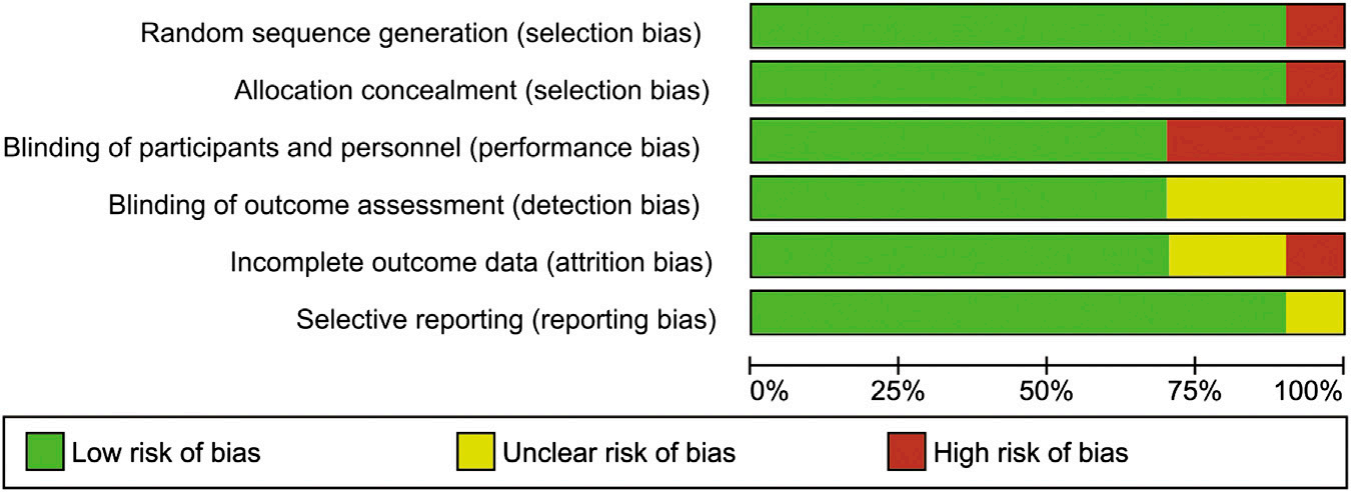
Risk of bias graph for included studies.

**FIGURE 3:**
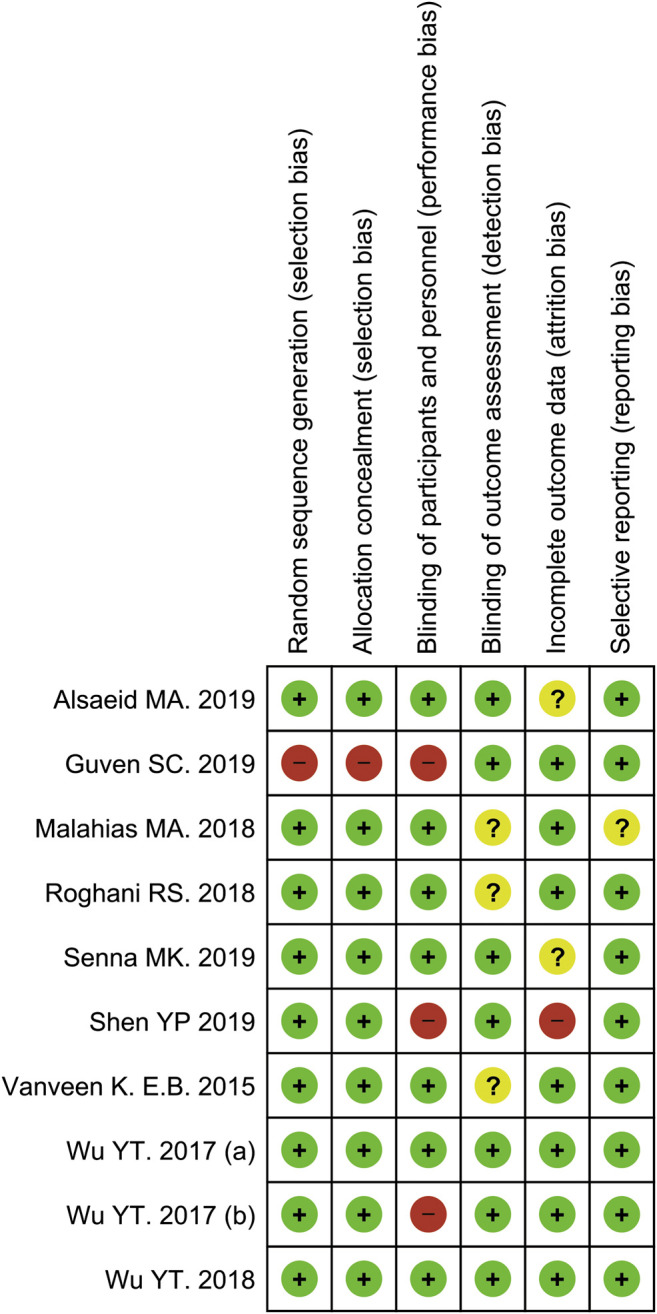
Risk of bias summary for each included study.

The methodological quality assessments of the included studies were revealed with the Cochrane risk of bias 2.0 tool. In this analysis, two studies were classified as low risk of bias (Wu et al., 2017b; Wu et al., 2018), three studies yielded a high risk of bias (Wu et al., 2017a; Güven et al., 2019; Shen et al., 2019), with the remaining five studies had an unclear risk of bias (van Veen et al., 2015; Malahias et al., 2018; Roghani et al., 2018; Alsaeid, 2019; Senna et al., 2019). Details of the quality assessment by the Cochrane risk of bias 2.0 tool is presented in Figures 2, 3.

### Effect on Pain Intensity (VAS)

To measure pain intensity, a visual analog scale (VAS) was used in six studies Wu et al., 2017a; Wu et al., 2017b; Wu et al., 2018; Roghani et al.,2018; Malahias et al.,2018, and Senna et al.,2019) (Malahias et al., 2018; Roghani et al., 2018; Wu et al., 2017a; Wu et al., 2017b; Wu et al., 2018). Two studies by Wu et al., 2017b and Malahias et al., 2018 used hydrodissection with normal saline as a control group (Malahias et al., 2018; Wu et al., 2017b). A study by Wu et al., 2017b compared D5W with normal saline as a control group (Wu et al., 2017b). A study by Malahias et al. compared PRP with normal saline as a control group (Malahias et al., 2018). Both studies showed greater VAS reduction in the intervention group, however, the difference between groups was not significant in a Malahias et al. study (*p* = 0.09) (Malahias et al., 2018). In a study by Wu et al., 2017b, there was a significant VAS reduction between both groups (D5W *vs* NSS) at all follow up time points at 1, 3,6 months (mean differences: −2.07, 95% CI = −1.15 to −2.99, *p* < 0.001 at one month; −3.1, 95% CI = −2.25 to −3.95, *p* < 0.001 at 3 months; −4.24, 95% CI = −3.39 to −5.09, *p* < 0.001 at 6 months) (Wu et al., 2017b). A study by Wu et al., 2017a that compared PRP vs splinting as a control group, showed significantly greater VAS reduction in PRP group at 6 months (mean difference: −4.53, 95% CI = −3.91 to −5.15, *p* < 0.001). Both groups showed significant VAS reduction at all follow-up time points at 1, 3, 6 months (Wu et al., 2017a). A study by Wu et al., 2018 compared D5W with triamcinolone, showed significantly greater VAS reduction in the D5W group at four months (mean difference: −3.6, 95% CI = −2.6 to −4.5, *p* < 0.001) and six months (mean difference: −4.3, 95% CI = −3.2 to −5.4, *p* < 0.001) with the greatest difference between group observed at six months (Wu et al., 2018). A study by Senna et al. compared PRP with methylprednisolone showed significantly lower average VAS in the PRP group at 3 months follow-up (mean difference: −46.3, 95% CI = −43.62 to −48.98, *p* < 0.001) see Table 2 for p between groups (Senna et al., 2019). Both groups showed significant VAS reduction at all follow-up time points at 1, 3, 4, 6 months (Wu et al., 2018). A study by Roghani et al. compared two different doses of steroids (80 mg vs 40 mg triamcinolone) vs local anesthetics (2% lidocaine) as a control group, showed no significant VAS differences between groups at all follow-up time points at 2 weeks, 3, 6 months (Roghani et al., 2018). Nevertheless, each of the three groups showed a significant VAS reduction within-group at all follow-up time points (Roghani et al., 2018).

**TABLE 2:** Clinical effects of perineural injectates classified by outcome.

Outcomes study (diagnosis)	Treatment allocation	F/U (Months)	Intervention			Comparison			*p-*value between groups	Treatment details	Summary
	Intervention		Mean	SD	*p-*value	Mean	SD	*p-*value		Intervention	
	Comparison									Comparison	
**VAS**											
Wu et al., 2017a (mild to moderate CTS)	PRP Splint	Baseline	6.50	1.64	-	6.29	1.70	-	0.630	PRP 3 ml Neutral, 8 h overnight daily	Significant reduction PRP > splint at 6 months
		1 month	3.89	1.53	<0.001	3.88	1.53	<0.001	0.540		
		3 months	2.91	0.40	<0.001	3.36	1.42	<0.001	0.104		
		6 months	1.97	0.40	<0.001	2.99	1.48	<0.001	**0.018**		
Wu et al., 2017b (mild to moderate CTS)	D5W NSS	Baseline	6.67	1.64	-	6.56	1.64	-	0.810	D5W 5 ml NSS 5 ml	Significant reduction D5W > NSS at all F/U time points
		1 month	4.60	1.91	<0.001	5.64	1.91	0.002	**0.001**		
		3 months	3.57	1.64	<0.001	4.70	2.52	<0.001	**0.020**		
		6 months	2.43	1.64	<0.001	4.59	2.52	<0.001	**<0.001**		
Wu et al., 2018 (mild to moderate CTS)	D5W Triamcinolone	Baseline	6.30	1.56	-	6.20	1.04	-	0.743	D5W 5 ml Triamcinolone (10 mg/ml) 3 ml + NSS 2 ml	Significant reduction D5W > triamcinolone at 4 and 6 months
		1 month	4.20	1.56	<0.001	4.20	2.08	<0.001	NA		
		3 months	3.30	1.04	<0.001	3.60	1.56	<0.001	NA		
		4 months	2.80	1.56	<0.001	3.90	1.56	<0.001	**<0.01**		
		6 months	2.00	1.56	<0.001	4.50	2.08	<0.001	**<0.001**		
Roghani et al., 2018 (moderate CTS, Age > 50 years)	Triamcinolone 80 mg (intervention group I) Triamcinolone 40 mg (intervention group II) Lidocaine (Comparison)	GI: Baseline	7.29	2.05	-	5.80	1.88	-	NA	GI: Triamcinolone (40 mg/ml) 2 ml + 2% lidocaine 1 ml GII: Triamcinolone (40 mg/ml) 1 ml + 2% lidocaine 2 ml GIII: 2% lidocaine 3 ml	No significant difference between groups at all F/U time points, significant baseline VAS difference between GI and Comparison
		GI: 2 weeks	4.24	2.09	<0.001	4.20	1.75	<0.001	Not Sig		
		GI: 3 months	4.15	2.21	<0.001	3.19	2.12	<0.001	Not Sig		
		GI: 6 months	2.43	1.93	<0.001	2.75	2.56	<0.001	Not Sig		
		GII: Baseline	6.22	2.74	-						
		GII: 2 weeks	4.81	2.39	<0.001						
		GII: 3 months	3.23	2.03	<0.001						
		GII: 6 months	2.00	1.44	<0.001						
Malahias et al., 2018 (mild to moderate CTS)	PRP NSS	Baseline	67.88	29.20	-	53.98	27.86	-	NA	PRP 2 ml NSS 2 ml	No significant difference between groups at all F/U time points
		1 month	NA	NA	NA	NA	NA	NA	0.164		
		3 months	NA	NA	NA	NA	NA	NA	0.090		
Senna et al., 2019 (mild to moderate CTS)	PRP Methylprednisolone	Baseline	68.10	6.00	-	69.50	4.90	-	0.242	PRP 2 ml Methylprednisolone (40 mg/ml) 1 ml	Significantly lower average VAS of PRP group at 3 months
		1 month	24.40	7.30	Sig	25.90	8.30	Sig	0.737		
		3 months	21.80	6.50	Sig	25.20	7.10	Sig	**0.040**		
**BCTQs (55)**											
Wu et al., 2017a (mild to moderate CTS)	PRP Splint	Baseline	26.17	6.02	-	24.93	6.68	-	0.457	PRP 3 ml Neutral, 8 h overnight daily	Significant improvement PRP > splint at 3 and 6 months
		1 month	17.17	3.45	<0.001	18.43	5.26	<0.001	0.098		
		3 months	15.76	2.74	<0.001	18.13	5.59	<0.001	**0.017**		
		6 months	14.14	2.46	<0.001	16.20	4.71	<0.001	**0.045**		
Wu et al., 2017b (mild to moderate CTS)	D5W NSS	Baseline	30.20	6.84	-	28.07	10.57	-	0.360	D5W 5 ml NSS 5 ml	Significant improvement D5W > NSS at all F/U time points
		1 month	20.83	5.80	<0.001	22.37	9.64	<0.001	**0.020**		
		3 months	17.60	4.38	<0.001	20.50	11.06	<0.001	**0.010**		
		6 months	15.30	3.29	<0.001	21.60	11.28	0.002	**<0.001**		
Wu et al., 2018 (mild to moderate CTS)	D5W Triamcinolone	Baseline	28.20	6.24	-	27.60	7.27	-	0.723	D5W 5 ml Triamcinolone (10 mg/ml) 3 ml + NSS 2 ml	Significant improvement D5W > triamcinolone at 3 and 6 months
		1 month	19.80	4.68	<0.001	22.50	8.83	0.016	NA		
		3 months	16.40	3.64	<0.001	19.80	6.24	<0.001	NA		
		4 months	15.90	3.12	<0.001	21.20	6.75	0.002	**<0.010**		
		6 months	14.70	3.12	<0.001	23.70	8.31	0.128	**<0.001**		
**BCTQs (1–5)**											
Alsaeid, 2019 (mild to moderate CTS)	Hyaluronidase (H) Dexamethasone (D)	Baseline	2.7	0.1	-	2.8	0.2	-	0.456	Hyaluronidase 300 IU in NSS 2 ml + 0.5% bupivacaine 3 ml dexamethasone (4 mg/ml) 2 ml + 0.5% bupivacaine 3 ml	Significant improvement H > D at all F/U time points
		1 week	1.6	0.2	<0.05	2	0.1	<0.05	**<0.05**		
		1 month	1.4	0.3	0.023	1.9	0.2	<0.05	**0.029**		
		3 months	1.3	0.2	0.041	1.7	0.3	0.012	**0.047**		
		6 months	1.7	0.5	<0.05	2.7	0.3	0.213	**<0.05**		
Güven et al., 2019 (mild to moderate CTS)	PRP + splint Splint	Baseline	3.00	0.7	-	2.3	0.6	-	**0.001**	PRP 1 ml + overnight daily wrist splint splining	Significant improvement in PRP + splinting group
		1 month	1.7	0.6	<0.001	1.6	0.5	<0.001	**0.009**		
Senna et al., 2019 (mild to moderate CTS)	PRP Methylprednisolone	Baseline	3.5	0.4	-	3.4	0.4	-	0.274	PRP 2 ml Methylprednisolone (40 mg/ml) 1 ml	Significant improvement PRP > Methylprednisolone at 3 months
		1 month	2.4	0.6	Sig	2.5	0.5	Sig	0.790		
		3 months	2.0	0.7	Sig	2.4	0.7	Sig	**0.007**		
Shen et al., 2019 (moderate CTS	PRP D5W	Baseline	2.5	1.02	-	2.4	0.51	-	0.876	PRP 2 ml D5W 3 ml	No significant difference between groups
		1 month	1.6	0.51	<0.001	1.8	0.51	<0.001	0.883		
		3 months	1.4	0.00	<0.001	1.6	0.51	<0.001	0.480		
		6 months	1.3	0.00	<0.001	1.4	0.51	<0.001	0.447		
**BCTQf (40)**											
Wu et al., 2017a (mild to moderate CTS)	PRP Splint	Baseline	19.23	5.91	-	18.13	3.56	-	0.387	PRP 3 ml Neutral, 8 h overnight daily	Significant improvement PRP > splint at all F/U time points
		1 month	12.24	3.01	<0.001	14.40	3.83	0.001	**0.002**		
		3 months	10.79	2.19	<0.001	13.63	1.97	<0.001	**<0.001**		
		6 months	10.41	2.63	<0.001	12.93	3.56	<0.001	**0.001**		
Wu et al., 2017b (mild to moderate CTS)	D5W NSS	Baseline	21.87	3.77	-	19.93	5.26	-	0.11	D5W 5 ml NSS 5 ml	Significant improvement D5W > NSS at all F/U time points
		1 month	14.17	3.94	<0.001	18.00	5.75	0.09	**<0.001**		
		3 months	12.90	2.84	<0.001	16.77	6.46	0.005	**<0.001**		
		6 months	11.43	2.51	<0.001	17.07	6.74	0.03	**<0.001**		
Wu et al., 2018 (mild to moderate CTS)	D5W Triamcinolone	Baseline	20.70	5.76	-	19.70	4.16	-	-	D5W 5 ml Triamcinolone (10 mg/ml) 3 ml + NSS 2 ml	Significant improvement D5W > triamcinolone at 4 and 6 months
		1 month	15.00	4.16	<0.001	16.10	5.20	0.008	NA		
		3 months	12.90	2.60	<0.001	15.00	4.16	<0.001	NA		
		4 months	12.20	3.12	<0.001	15.90	4.16	0.002	**<0.001**		
		6 months	11.40	2.08	<0.001	16.60	4.16	0.063	**<0.001**		
**BCTQf (1–5)**											
Alsaeid, 2019 (mild to moderate CTS)	Hyaluronidase (H) Dexamethasone (D)	Baseline	2.6	0.4	-	2.7	0.3	-	0.243	Hyaluronidase 300 IU in NSS 2 ml + 0.5%bupivacaine 3 ml, dexamethasone (4 mg/ml) 2 ml+ 0.5% bupivacaine 3 ml	Significant improvement of H > D at all follow up time points
		1 week	1.4	0.4	0.045	1.9	0.2	0.01	**0.046**		
		1 month	1.1	0.3	<0.05	1.8	0.1	0.034	**<0.05**		
		3 months	1.0	0.6	0.037	1.8	0.3	<0.05	**0.019**		
		6 months	1.8	0.4	0.028	2.6	0.1	0.2	**0.033**		
Güven et al., 2019 (mild to moderate CTS)	PRP + splint Splint	Baseline	2.7	0.8	-	2.2	0.6	-	**0.026**	PRP 1 ml + overnight daily wrist splint	Significant improvement in PRP + splinting group
		1 month	1.8	0.6	<0.001	1.7	0.6	0.001	**0.018**		
Senna et al., 2019 (mild to moderate CTS)	PRP Methylprednisolone	Baseline	3.5	0.4	-	3.4	0.5	-	0.204	PRP 2 ml Methylprednisolone (40 mg/ml) 1 ml	Significant improvement PRP > methylprednisolone group at 3 months
		1 month	3.1	0.4	Sig	3.0	0.4	Sig	0.203		
		3 months	2.1	0.6	Sig	2.5	0.6	Sig	**0.002**		
Shen et al., 2019 (moderate CTS)	PRP D5W	Baseline	2.5	0.51	-	2.5	1.02	-	NA	PRP 2 ml D5W 3 ml	Significant improvement PRP > D5W at 3 months
		1 month	1.7	0.51	<0.001	1.8	0.51	<0.001	0.484		
		3 months	1.4	0.00	<0.001	1.7	0.51	<0.001	**0.044**		
		6 months	1.3	0.51	<0.001	1.5	0.51	<0.001	0.267		
**BCTQ combined**											
Roghani et al., 2018 (moderate CTS, Age > 50 years)	Triamcinolone 80 mg (group I) Triamcinolone 40 mg (group II) Lidocaine (Comparison)	GI: Baseline	55.81	15.04	-	45.22	13.84	-	NA	GI: Triamcinolone (40 mg/ml) 2 ml + 2% lidocaine 1 ml GII: Triamcinolone (40 mg/ml) 1 ml + 2% lidocaine 2 ml Control: 2% lidocaine 3 ml	No significant difference between groups at all F/U time points
		GI: 2 weeks	41.95	11.26	0.001	40.45	11.08	0.018	NA		
		GI: 3 months	40.43	12.14	0.001	41.27	12.65	0.018	NA		
		GI: 6 months	34.06	10.25	0.001	36.94	13.04	0.018	NA		
		GII: Baseline	47.70	11.70	-						
		GII: 2 weeks	44.94	09.70	<0.001						
		GII: 3 months	43.41	10.97	<0.001						
		GII: 6 months	38.67	11.21	<0.001						
**Q-DASH success ratio**											
Malahias et al., 2018 (mild to moderate CTS)	PRP NSS	Baseline	NA	NA	-	NA	NA	-	NA	PRP 2 ml NSS 2 ml	Significant different PRP > NSS at 3 months
		1 month	NA	NA	NA	NA	NA	NA	NA		
		3 months	NA	NA	NA	NA	NA	NA	**0.011**		
**Q-DASH decrease**											
Malahias et al., 2018 (mild to moderate CTS)	PRP NSS	Baseline	NA	NA	-	NA	NA	-	NA	PRP 2 ml NSS 2 ml	Significant different PRP > NSS at 3 months
		1 month	NA	NA	NA	NA	NA	NA	NA		
		3 months	NA	NA	NA	NA	NA	NA	**0.022**		
**Subjective symptom change**		**F/U At 3** **months**	N (30)	%		N (25)	%			1 ml of methylprednisolone 40 mg with lidocaine 10 mg NSS 1 ml	No significant difference between 2 groups
Van Veen et al., 2015, UNE	Methylprednisolone NSS	Complete recovery	2	7		1	4		0.871		
		Clear improvement	3	10		4	16				
		Some improvement	4	13		2	8				
		No improvement	20	67		17	17				
		Some worsening	1	3		1	1				
		Clear worsening	0	0		0	0				
**Global assessment of treatment results**		**F/U at3and/or 6 months**									
Wu et al., 2017b, (mild to moderate CTS)	D5W NSS	**At 3** **months**	N (30)	%		N (30)	%			D5W 5 ml NSS 5 ml	Significant improvement D5W > NSS at 6 months
		Improved	21	70		15	50		0.11		
		No change	9	30		15	50				
		**At 6 months**	N (30)	%		N (30)	%				
		Improved	23	76		12	40		**0.004**		
		No change	7	24		18	60				
Wu et al., 2018 Mild to moderate CTS	D5W Triamcinolone	**At 6 months**	N (27)	%		N (27)	%			D5W 5 ml Triamcinolone (10 mg/ml) 3 ml + NSS 2 ml	Significant improvement D5W > triamcinolone
		Improved	24	88		10	37		**<0.001**		
		No change	3	12		17	63				
**Finger pinch strength (kg)**											
Wu et al., 2017a (mild to moderate CTS)	PRP Splint	Baseline	3.27	1.53	-	3.74	0.60	-	0.133	PRP 3 ml Neutral, 8 h overnight daily	No significant difference between groups at all F/U time points
		1 month	4.06	1.48	0.002	4.26	0.99	0.071	0.384		
		3 months	4.13	1.59	<0.001	4.22	0.93	0.040	0.138		
		6 months	4.45	1.26	<0.001	4.68	1.26	0001	0.482		
Monofilament (0–15)*											
Güven et al., 2019 (mild to moderate CTS)	PRP + splint Splint	Baseline	12.5	2.3	-	13.2	1.4	-	0.583	PRP 1 ml + overnight daily wrist splint Splint	No significant difference between groups at all F/U time points
		1 month	13.8	1.1	0.003	13.5	1.5	0.270	0.461		
**Static 2PD testing score (mm)**											
Güven et al., 2019 (mild to moderate CTS)	PRP + splint Splint	Baseline	3.3	1.1	-	3.0	0.7	-	0.512	PRP 1 ml + overnight daily wrist splint Splint	No significant difference between groups at all F/U time points
		1 month	2.7	0.8	0.002	2.6	0.8	0.019	0.862		
**Dynamic 2PD testing score (mm)**											
Güven et al., 2019 (mild to moderate CTS)	PRP + splint Splint	Baseline	3.2	1.2	-	2.8	0.8	-	0.301	PRP 1 ml + overnight daily wrist splint Splint	No significant difference between groups at all F/U time points
		1 month	2.4	0.7	0.004	2.6	0.8	0.212	0.583		
**Paresthesia**											
Senna et al., 2019 (mild to moderate CTS)	PRP Methylprednisolone	Baseline	39	90.7%	-	37	88.1%	-	0.697	PRP 2 ml Methylprednisolone (40 mg/ml) 1 ml	Significant improvement PRP > Methylprednisolone at 3 months
		1 month	8	18.6%	Sig	9	21.4%	Sig	0.745		
		3 months	4	9.3%	Sig	11	26.2%	Sig	**0.041**		
		
**Signs + ve Phalen’s test**											
Senna et al., 2019 (mild to moderate CTS)	PRP Methylprednisolone	Baseline	42	97.7%	-	40	95.2%	-	0.616	PRP 2 ml Methylprednisolone (40 mg/ml) 1 ml	Significant improvement PRP > Methylprednisolone at 3 months
		1 month	8	18.6%	Sig	9	21.4%	Sig	0.745		
		3 months	4	9.3%	Sig	11	26.2%	Sig	**0.041**		
	
**Signs + ve Tinel’s test**											
Senna et al., 2019 (mild to moderate CTS)	PRP Methylprednisolone	Baseline	34	79.1%	-	36	85.7%	-	0.422	PRP 2 ml Methylprednisolone (40 mg/ml) 1 ml	Significant improvement PRP > Methylprednisolone at 3 months
		1 month	6	14.0%	Sig	6	14.3%	Sig	0.745		
		3 months	2	4.7%	Sig	8	19%	Sig	**0.039**		
**EDS:MNCV (m/s)**											
Van Veen et al., 2015, UNE	Methylprednisolone NSS	MNCV across elbow (m/s)								1 ml of methylprednisolone 40 mg with lidocaine 10 mg NSS 1 ml	No significant change in both groups
		Baseline			NA	46.2	NA	NA	NA		
		3 months			NA	50.3	NA	NA	NA		
		MNCV slowing across elbow (m/s)									
		Baseline	11.7	NA	NA	11.2	NA	NA	NA		
		3 months	8.8	NA	NA	7.0	NA	NA	NA		
**EDS: SNCV (m/s)**											
Wu et al., 2017a (mild to moderate CTS)	PRP Splint	Baseline	30.18	7.07	-	32.35	6.07	-	0.205	PRP 3 ml Neutral, 8 h overnight daily	No significant differences between groups at all F/U time points
		1 month	32.45	6.85	<0.001	34.74	6.63	<0.001	0.779		
		3 months	32.82	6.96	<0.001	35.05	7.01	<0.001	0.917		
		6 months	33.92	7.34	<0.001	36.17	7.31	<0.001	0.925		
Wu et al., 2017b (mild to moderate CTS)	D5W NSS	Baseline	33.76	5.53	-	33.83	4.93	-	0.960	D5W 5 ml NSS 5 ml	Significant improvement D5W > NSS at all F/U time points
		1 month	35.46	6.41	0.040	34.08	4.98	0.990	**0.030**		
		3 months	36.29	5.81	0.003	33.72	5.64	0.990	**0.001**		
		6 months	36.75	6.52	0.004	34.08	5.70	0.990	**0.003**		
Wu et al., 2018 (mild to moderate CTS)	D5W Triamcinolone	Baseline	32.3	5.72	-	32.7	6.75	-	0.837	D5W 5 ml Triamcinolone (10 mg/ml) 3 ml + NSS 2 ml	No significant between groups at all F/U time points
		1 month	34.2	6.24	0.024	34.7	7.27	<0.001	0.850		
		3 months	34.6	6.24	0.004	35.4	7.27	<0.001	0.512		
		6 months	34.9	6.76	0.023	33.9	6.75	0.345	0.203		
Alsaeid, 2019 (mild to moderate CTS)	(H) (D)	Baseline	31.10	0.4	-	30.10	0.3	-	0.310	Hyaluronidase 300 IU in NSS 2 ml + 0.5%bupivacaine 3 ml, dexamethasone (4 mg/ml) 2 ml + 0.5% bupivacaine 3 ml	Significant improvement between groups (H > D) at all F/U time points
		1 week	32.90	0.1	0.039	31.40	0.4	<0.050	**<0.050**		
		1 month	32.50	0.6	0.022	31.90	0.6	0.042	**0.011**		
		3 months	32.70	0.4	<0.05	32.00	0.7	0.490	**0.048**		
		6 months	32.20	0.9	0.041	30.02	0.2	0.120	**<0.05**		
Güven et al., 2019 (mild to moderate CTS)	PRP + splint Splint	Baseline	40.90	6.50	-	42.40	5.10	-	0.369	PRP 1 ml + overnight daily wrist splint Splint	Significant improvement in PRP + splinting group
		1 month	43.4	5.70	0.001	42.90	4.70	0.228	**0.026**		
Senna et al., 2019 (mild to moderate CTS)	PRP Methylprednisolone	Baseline	32.2	1.9	-	31.4	2.2	-	0.080	PRP 2 ml Methylprednisolone (40 mg/ml) 1 ml	No significant differences between groups at all F/U time points
		1 month	34.9	2.5	<0.001	34.2	2.5	<0.001	0.205		
		3 months	35.7	3.6	<0.001	34.3	2.8	<0.001	0.049		
Shen et al., 2019 (moderate CTS	PRP D5W	Baseline	27.80	7.14	-	30.00	6.63	-	0.309	PRP 2 ml D5W 3 ml	No significant differences between groups at all F/U time points
		1 month	29.10	3.12	0.029	31.30	6.63	0.020	0.854		
		3 months	30.00	6.63	0.001	31.50	6.63	0.125	0.244		
		6 months	30.60	7.65	0.004	31.20	7.14	0.627	0.099		
**EDS: DML (ms)**											
Wu et al., 2017a (mild to moderate CTS)	PRP Splint	Baseline	5.66	1.48	-	5.21	1.26	-	0.215	PRP 3 ml Neutral, 8 h overnight daily	No significant differences between groups at all F/U time points
		1 month	5.28	1.26	<0.001	4.96	1.20	0.041	0.199		
		3 months	5.26	1.37	0.006	4.98	1.20	0.016	0.157		
		6 months	5.18	1.42	0.001	4.74	1.04	<0.001	0.934		
Wu et al., 2017b (mild to moderate CTS)	D5W NSS	Baseline	4.89	1.31	-	4.68	0.82	-	0.450	D5W 5 ml NSS 5 ml	Significant improvement D5W > NSS at 1 and 3 months follow- up
		1 month	4.68	1.26	0.220	4.72	0.82	0.990	**0.040**		
		3 months	4.64	1.20	0.200	4.72	0.82	0.990	**0.030**		
		6 months	4.53	1.10	0.430	4.64	0.88	0.990	0.120		
Wu et al., 2018 (mild to moderate CTS)	D5W Triamcinolone	Baseline	5.20	1.56	-	5.4	1.56	-	0.698	D5W 5 ml Triamcinolone (10 mg/ml) 3 ml + NSS 2 ml	No significant differences between groups at all F/U time points
		1 month	5.00	1.56	0.184	5.0	1.04	<0.001	0.253		
		3 months	4.80	1.04	0.030	4.9	1.04	0.022	0.792		
		6 months	4.80	1.04	0.307	5.0	1.56	0.356	0.828		
Roghani et al., 2018 (moderate CTS, Age > 50 years)	Triamcinolone 80 mg (group I) Triamcinolone 40 mg (group II) Lidocaine (Comparison)	GI: Baseline	5.08	1.35	-	4.69	1.51	-	NA	GI: Triamcinolone (40 mg/ml) 2 ml + 2% lidocaine 1 ml GII: Triamcinolone (40 mg/ml) 1 ml + 2% lidocaine 2 ml Control I: 2% lidocaine 3 ml	No significant differences between groups at all F/U time points
		GI: 2 weeks	4.70	1.20	0.001	4.50	1.32	0.887	Not sig		
		GI: 3 months	5.00	1.12	0.001	4.45	1.19	0.887	Not sig		
		GI: 6 months	4.55	0.66	0.001	4.16	0.70	0.887	Not sig		
		GII: Baseline	5.15	1.23	-						
		GII: 2 weeks	4.80	1.23	<0.001						
		GII: 3 months	4.32	1.23	<0.001						
		GII: 6 months	4.65	0.80	<0.001						
Alsaeid, 2019 (mild to moderate CTS)	(H) (D)	Baseline	4.80	0.70	-	4.90	0.50	-	0.740	Hyaluronidase 300 IU in NSS 2 ml + 0.5%bupivacaine 3 ml, dexamethasone (4 mg/ml) 2 ml+ 0.5% bupivacaine 3 ml	Significant improvement H > D at all follow-up time points
		1 week	4.10	0.10	<0.050	4.50	0.40	0.044	**0.024**		
		1 month	3.70	0.70	<0.050	4.10	0.60	<0.050	**<0.050**		
		3 months	3.50	0.20	0.030	4.00	0.30	0.012	**0.036**		
		6 months	3.90	0.80	<0.050	4.80	0.70	0.450	**0.029**		
Güven et al., 2019 (mild to moderate CTS)	PRP + splint Splint	Baseline	4.80	0.80	-	4.5	0.70	-	0.314	PRP 1 ml + overnight daily wrist splint Splint	Significant improvement in PRP + splinting group
		1 month	4.40	0.70	<0.001	4.5	0.60	0.273	**0.005**		
Senna et al., 2019 (mild to moderate CTS)	PRP Methylprednisolone	Baseline	4.90	0.90	-	5.00	0.70	-	0.613	PRP 2 ml Methylprednisolone (40 mg/ml) 1 ml	No significant differences between groups at all F/U time points
		1 month	4.50	0.60	<0.001	4.60	0.60	<0.001	0.342		
		3 months	4.40	0.60	<0.001	4.50	0.80	<0.001	0.559		
Shen et al., 2019 (moderate CTS	PRP D5W	Baseline	5.80	1.53	-	5.50	1.53	-	0.714	PRP 2 ml D5W 3 ml	Significant improvement PRP > D5W at 6 months
		1 month	5.60	1.53	0.281	5.40	1.53	1.000	0.633		
		3 months	5.40	1.53	0.117	5.40	1.53	1.000	0.240		
		6 months	5.40	1.53	0.112	5.30	1.53	1.000	**0.028**		
											
**EDS: Motor conduction (m/s)**											
Senna et al., 2019 (mild to moderate CTS)	PRP Methylprednisolone	Baseline	56.3	2.3	-<0.001	57.1	3.2	-	0.131	PRP 2 ml Methylprednisolone (40 mg/ml) 1 ml	Significant improvement PRP > methylprednisolone at 3 months
		1 month	57.1	1.9	<0.001	59.7	3.6	<0.001	0.082		
		3 months	57.4	3.5		59.9	3.7	<0.001	**0.002**		
**EDS: Distal CMAP amplitude (mV)**											
Senna et al., 2019 (mild to moderate CTS)	PRP Methylprednisolone	Baseline	5.8	1.4	-	6.4	1.7	-	0.088	PRP 2 ml Methylprednisolone (40 mg/ml) 1 ml	No significant differences between groups at all F/U time points
		1 month	8.6	2.1	<0.001	9.3	3	<0.001	0.281		
		3 months	8.8	2.2	<0.001	9.5	3	<0.001	0.313		
**EDS: Sensory latency (ms)**											
Senna et al., 2019 (mild to moderate CTS)	PRP Methylprednisolone	Baseline	5.2	0.5	-	4.9	0.5	-	0.068	PRP 2 ml Methylprednisolone (40 mg/ml) 1 ml	Significant improvement PRP > methylprednisolone at 3 months
		1 month	4.2	0.8	<0.001	4.1	0.6	<0.001	0.537		
		3 months	3.8	0.8	<0.001	4.1	0.7	<0.001	**0.037**		
**EDS: SNAP amplitude (mV)**											
Senna et al., 2019 (mild to moderate CTS)	PRP Methylprednisolone	Baseline	16.3	1.8	-	17	1.7	-	0.077	PRP 2 ml Methylprednisolone (40 mg/ml) 1 ml	No significant differences between groups at all F/U time points
		1 month	19.1	2.3	<0.001	19.7	2.3	<0.001	0.239		
		3 months	18.5	2.2	<0.001	19.2	2.2	<0.001	0.120		
**CSA (mm** ^**2**^ **)**											
VanVeen et al., 2015, UNE	Methylprednisolone NSS	Baseline	11.9	-	-	13.2	-	-	NA	1 ml of methyl-prednisolone 40 mg with lidocaine 10 mg NSS 1 ml	No significant difference between groups
		3 months	10.9	-	0.043	13.2	-	NA	NA		
Wu et al., 2017a (mild to moderate CTS)	PRP Splint	Baseline	14.01	4.49	-	12.91	4.43	-	0.343	PRP 3 ml Neutral, 8 h overnight daily	Significant differences PRP > splint at all follow-up time points
		1 month	11.86	4.16	<0.001	11.72	4.44	<0.001	**0.004**		
		3 months	11.35	4.05	<0.001	11.23	3.94	<0.001	**0.003**		
		6 months	10.93	4.10	<0.001	10.87	4.16	<0.001	**0.004**		
Wu et al., 2017b (mild to moderate CTS)	D5W NSS	Baseline	12.36	1.92	-	12.29	1.97	-	0.890	D5W 5 ml NSS 5 ml	Significant differences D5W > NSS at 3 and 6 months
		1 month	11.00	1.80	<0.001	11.32	2.02	<0.001	0.090		
		3 months	10.53	1.70	<0.001	11.22	2.02	<0.001	**0.010**		
		6 months	10.26	1.92	<0.001	11.11	2.08	<0.001	**0.001**		
Wu et al., 2018 (mild to moderate CTS)	D5W Triamcinolone	Baseline	12.7	2.60	-	13.0	3.11	-	0.613	D5W 5 ml Triamcinolone (10 mg/ml) 3 ml + NSS 2 ml	No significant difference between groups
		1 month	11.3	2.60	<0.001	11.2	2.60	<0.001	0.170		
		3 months	10.8	2.08	<0.001	10.8	2.60	<0.001	0.346		
		6 months	10.5	2.60	<0.001	11.4	3.12	0.003	0.298		
Roghani et al., 2018 (moderate CTS, Age > 50 years)	Triamcinolone 80 mg (group I) Triamcinolone 40 mg (group II) Lidocaine (Control)	GI: Baseline	11.73	2.53	-	12.09	3.96	-	**NA**	GI: Triamcinolone (40 mg/ml) 2 ml + 2% lidocaine 1 ml GII: Triamcinolone (40 mg/ml) 1 ml + 2% lidocaine 2 ml GIII: 2% lidocaine 3 ml	No significant difference between groups
		GI: 2 weeks	10.77	2.49	0.002	11.23	2.72	0.007	**0.512**		
		GI: 3 months	10.78	2.18	0.002	11.37	1.97	0.007	**0.512**		
		GI: 6 months	10.45	2.40	0.002	10.76	2.05	0.007	**0.512**		
		GII: Baseline	12.23	2.39	-						
		GII: 2 weeks	11.55	2.19	<0.001						
		GII: 3 months	11.26	2.37	<0.001						
		GII: 6 months	10.26	2.34	<0.001						
Alsaeid, 2019 (mild to moderate CTS)	Hyaluronidase (H) Dexamethasone (D)	Baseline	13.00	0.50	-	13.20	0.80	-	0.210	Hyaluronidase 300 IU in NSS 2 ml + 0.5% bupivacaine 3 ml dexamethasone (4 mg/ml) 2 ml+ 0.5% bupivacaine 3 ml	Significant differences H > D at all follow-up time points
		1 week	12.60	0.10	<0.050	12.50	0.30	0.012	**<0.050**		
		1 month	12.10	0.20	0.300	12.30	0.90	<0.050	**0.045**		
		3 months	12.00	0.50	0.023	12.10	0.70	0.019	**<0.050**		
		6 months	12.40	0.70	0.310	12.90	0.80	<0.050	**0.034**		
Güven et al., 2019 (mild to moderate CTS)	PRP + splint Splint	Baseline	14.10	4.9	-	11.50	2.00	-	0.081	PRP 1 ml + overnight daily wrist splint	No significant difference between groups
		1 month	12.60	4.5	0.003	10.90	2.20	0.026	0.414		
Senna et al., 2019 (mild to moderate CTS)	PRP Methylprednisolone	Baseline	13.60	1.20	-	13.20	1.30	-	0.215	PRP 2 ml Methylprednisolone (40 mg/ml) 1 ml	No significant difference between groups
		1 month	10.90	1.30	<0.001	11.20	1.60	<0.001	0.414		
		3 months	10.60	1.40	<0.001	10.90	1.70	<0.001	0.340		
Shen et al., 2019 (moderate CTS	PRP D5W	Baseline	14.50	3.06	-	13.90	2.04	-	0.286	PRP 2 ml D5W 3 ml	Significant improvement between groups (PRP > D5W) at 3rd and 6th-month assessments
		1 month	12.60	3.06	<0.001	12.60	2.04	0.002	0.538		
		3 months	11.60	2.55	<0.001	12.20	2.04	<0.001	**0.010**		
		6 months	11.20	2.55	<0.001	12.00	2.55	0.001	**0.018**		
**Delta CSA**											
Malahias et al., 2018 (mild to moderate CTS)	PRP NSS	Baseline	0.057	0.028	NA	0.052	0.035	NA	NA	PRP 2 ml NSS 2 ml	No significant difference between groups
		1 month	NA	NA	NA	NA	NA	NA	NA		
		3 months	0.041	0.019	NA	0.043	0.015	NA	0.132		

Delta CSA: Cross-sectional area difference of the median nerve’s surface at the tunnel’s inlet minus the median nerve’s surface proximal to the tunnel and overpronator quadrant.

Q-DASH success: Presented as the number of patients with either a. higher than 25% improvement in Q-DASH score at final follow-up or b. positive Q-DASH difference bigger than 8.0 points at final follow-up; divided by the number of patients in the belonging group.

Q-DASH decrease: Presented as the number of patients with a final Q-DASH score decreased greater than 8.0 points; divided the number of patients in the belonging group.

### Effect on Clinical Symptoms, Function, and Physical Performance

Standardized outcome measures specific for carpal tunnel syndrome and upper extremity disorders were used in nine carpal tunnel syndrome studies (Wu et al., 2017a; Wu et al., 2017b; Malahias et al., 2018; Roghani et al., 2018; Wu et al., 2018; Alsaeid, 2019; Güven et al., 2019; Senna et al., 2019; Shen et al., 2019). The Boston Carpal Tunnel Syndrome questionnaire (BCTQ) was used in eight studies, except Malahias et al. which used the Quick Disability of Arms Shoulders and Hands (Q-DASH) questionnaire. For the ulnar nerve entrapment study by vanVeen et al. the authors developed a 6-point subjective symptom change scoring system for patients to rate their symptoms (van Veen et al., 2015). Two studies, Wu et al., 2017b and Wu et al., 2018 added global assessment of treatment results as another patient-rated outcome measure (Wu et al., 2017b; Wu et al., 2018). Physical performance including finger pinch strength, monofilament test scores, static 2-point discrimination test (static 2PD) and dynamic 2-point discrimination test (dynamic 2PD), paresthesia symptoms, positive Tinel’s sign, and Phalen’s test. Finger pinch strength were measured by Wu et al. (Wu et al., 2017a). Monofilament test scores, static 2-point discrimination test (static 2PD), and dynamic two point discrimination test (dynamic 2PD) were measured by Güven et al. (Güven et al., 2019). Paresthesia symptoms, positive Tinel’s sign, and Phalen’s test were measured by Senna et al. (Senna et al., 2019).

#### Boston Carpal Tunnel Syndrome Questionnaire (BCTQ)

For BCTQs (55) outcomes, Two studies Wu et al., 2017b and Wu et al., 2018 used D5W as an intervention group (Wu et al., 2017b; Wu et al., 2018). A study by Wu et al., 2018 compared D5W with triamcinolone. (Wu et al., 2018). Another study (Wu et al., 2017b in 2017 compared D5W with normal saline (Wu et al., 2017b). Both studies showed greater results on BCTQs (55) in the intervention group. In a study by Wu et al., 2017b showed significant improvement BCTQs (55) in the D5W group at all follow up time points; at 1 month (mean difference: −9.37, 95% CI = −6.09 to −12.65, *p* < 0.001), 3 months (mean difference: −12.6, 95% CI = −9.63 to −15.57, *p* < 0.001), and 6 months (mean difference: −14.9, 95% CI = −12.13 to −17.67, *p* < 0.001) with difference between group observed after 3 months (Wu et al., 2017b). In the same way of another research by Wu et al., 2017a compared PRP with splinting as a control group, there was a significant improvement BCTQs (55) at 3 and 6 months (mean differences: −10.41, 95% CI = −7.99 to −12.83, *p* < 0.001 and −12.03, 95% CI = −9.65 to −14.41, *p* < 0.001) with the observed difference between groups after three months as well (Wu et al., 2018).

For BCTQs (1–5) outcomes, two studies (Alsaeid and Senna et al.) used corticosteroid medications (dexamethasone and methylprednisolone) as a comparison group (Alsaeid, 2019; Senna et al., 2019). A study by Alsaeid compared dexamethasone with hyaluronidase as an intervention group. This study showed significant BCTQs (1–5) improvement in hyaluronidase group at all follow up time points (mean differences were: −1.1, 95% CI = −0.99 to −1.20, *p* < 0.05 at 1 week; −1.3, 95% CI = −1.16 to −1.44, *p* = 0.023 at 1 month; −1.4, 95% CI = −1.29 to −1.50, *p* = 0.041 at 3 months; −1, 95% CI = −0.77 to −1.23, *p* < 0.05 at 6 months) (Alsaeid, 2019). Similarly in a Senna et al. study, which compared methylprednisolone with PRP, the result showed significant BCTQs (1–5) improvement in PRP group at 3 months (mean difference: −1.5, 95% CI = −1.26 to −1.74, *p* < 0.001) (Senna et al., 2019). Furthermore, in a study by Shen et al., using PRP compared with D5W, the results did not show significant difference of BCTQs (1–5) between groups at all follow up time points (Shen et al., 2019). This might imply that both PRP and D5W gave positive clinical symptom effect for moderate CTS.

For BCTQf (40) measurement, two studies by Wu et al., 2017b and Wu et al., 2018 used D5W as an intervention group (Wu et al., 2017b; Wu et al., 2018). A study by Wu et al., 2017b compared D5W with normal saline (Wu et al., 2017b). Another study by Wu et al., 2018 compared D5W with triamcinolone (Wu et al., 2018). Both studies showed positive result on D5W group in BCTQf (40), which presented significant BCTQf (40) improvement at 1, 3, 6 months (mean differences were: −6.99, 95% CI = −4.57 to −9.41, *p* < 0.001 at 1 month; −8.44, 95% CI = −6.14 to −10.74, *p* < 0.001 at 3 months; −8.82, 95% CI = -6.46 to −11.18, *p* < 0.001 at 6 months) and 4, 6 months (mean differences: −8.5, 95% CI −5.97 to −11.03, *p* < 0.001 at 4 months and −9.3, 95% CI = −6.93 to −11.67, *p* < 0.001 at 6 months) respectively (Wu et al., 2017b; Wu et al., 2018).

For BCTQf (1–5) measurement, Two studies (Alsaeid and Senna et al.) used corticosteroid medication (dexamethasone and methylprednisolone) as a comparison group (Alsaeid, 2019; Senna et al., 2019). A study by Alsaeid compared dexamethasone with hyaluronidase as an intervention group. This study showed significant BCTQf (1–5) improvement in hyaluronidase group at all follow up time points (mean differences: −1.2, 95% CI = −0.94 to −1.46, *p* = 0.045 at 1 week; −1.5, 95% CI = −1.27 to −1.73, *p* < 0.05 at 1 month; −1.6, 95% CI = −1.27 to −1.92, *p* = 0.037 at 3 months; −0.8, 95% CI = −0.54 to −1.06, *p* = 0.028 at 6 months) (Alsaeid, 2019). Similarly with Senna et al. which compared Methylprednisolone with PRP as an intervention group. The result showed significant positive effect on PRP in BCTQf (1–5) at 3 months (mean difference: −1.4, 95% CI = −1.18 to −1.62, *p* < 0.001) (Senna et al., 2019). Another PRP study, Güven et al. studied mild to moderate CTS, compared PRP plus splinting with splinting alone, delta analysis showed significantly greater improvement in PRP plus splinting group (*p* = 0.018) (Güven et al., 2019).

For BCTQ combined score (BCTQ combined), used in a study by Roghani et al. compared triamcinolone 80 mg, triamcinolone 40 mg, and lidocaine as a comparison group. The results did not show a significant difference between all three groups at all follow-up time points (Roghani et al., 2018).

For Q-DASH success ratio and Q-DASH decrease, the study by Malahias et al. used PRP as an intervention group, which compared with NSS as a comparison group. This results showed significantly greater improvement in PRP comparing to the NSS group at 3 months (Malahias et al., 2018).

#### Subjective Symptom Changes and Global Assessment of Treatment Results

In the subjective symptom change, the study by vanVeen et al. compared methylprednisolone with NSS as a comparison group, which did not present a significant difference between methylprednisolone and NSS groups at all follow-up time points (van Veen et al., 2015). On the other hand, for global assessment of treatment results. two studies by Wu et al., 2017b and Wu et al., 2018 used D5W as an intervention group. Each study compared D5W with NSS and triamcinolone, respectively. Both studies showed significantly greater improvement on a global assessment of treatment results in the D5W group at 6 months (Wu et al., 2017b; Wu et al., 2018).

#### Physical Performance

Two studies by Wu et al., 2017a and Güven et al. used splinting as a comparison group. The study by Wu et al., 2017a compared splinting with PRP as an intervention group, whose results did not show a significant difference between groups on finger pinch strength at all follow-up time points (Wu et al., 2017a). Similarly, a study by Güven et al., compared splinting with PRP as an intervention group. The result does not present a significant difference between groups on monofilament, static 2PD test, and dynamic 2PD test at all follow-up time points (Güven et al., 2019). Another study by Senna et al. used PRP as an intervention group, comparing with methylprednisolone. The results showed significant improvement on paresthesia (*p*-value between-group = 0.041), positive Phalen’s test (*p*-value between groups = 0.041), and positive Tinel’s sign (*p*-value between groups = 0.039) in PRP group at three months (Senna et al., 2019).

### Effect on an Electrodiagnostic Study (EDS)

Of the ten studies, nine studies in carpal tunnel syndrome patients had EDS performed on median nerves (Wu et al., 2017a; Wu et al., 2017b; Malahias et al., 2018; Roghani et al., 2018; Wu et al., 2018; Alsaeid, 2019; Güven et al., 2019; Senna et al., 2019; Shen et al., 2019). Only one study in cubital tunnel syndrome patients had EDS performed on the ulnar nerve (van Veen et al., 2015). Sensory nerve conduction velocity (SNCV) and distal motor latency (DML) are the most commonly evaluated parameters as they were evaluated in all nine carpal tunnel syndrome studies.

SNCV was measured in median nerve studies. Three of the studies, studied in mild to moderate CTS, showed significant improvement between groups at all follow-up time points. Wu et al., 2017b compared injectate with NSS as a control, showed significantly greater improvement in D5W than NSS group at 1, 3 and 6 months (mean differences: 1.70, 95% CI = 1.39 to 4.79, *p* = 0.040 at 1 month; 2.53, 95% CI = 0.40 to 5.46, *p* = 0.003 at 3 months; 2.99, 95% CI = 0.13 to 6.11, *p* = 0.004 at 6 months) (Wu et al., 2017b). In another study, Alsaeid compared injectate with injectate, showed significantly greater improvement in hyaluronidase group than dexamethasone group at 1 week, first, third, and sixth month (mean differences were: 1.80, 95% CI = 1.61 to 1.99, *p* = 0.039 at 1 week; 1.40, 95% CI = 1.07 to 1.73, *p* = 0.022 at 1 month; 1.60, 95% CI = 1.34 to 1.86, *p* < 0.050 at 3 months; 1.10, 95% CI = 0.65 to 1.55, *p* = 0.041 at 6 months) (Alsaeid, 2019). Güven et al. studied in mild to moderate CTS, compared PRP plus splinting with splinting alone, delta analysis showed significantly greater improvement in PRP plus splinting group (*p* = 0.026) (Güven et al., 2019).

DML was also measured in the median nerve study. Four of the studies showed significant improvement between groups. Wu et al., 2017b studied mild to moderate CTS, compared D5W with NSS as a control, showed significantly greater improvement in dextrose group than NSS group at 1 and 3 months (mean differences were: −0.21, 95% CI = −0.45 to −0.87, *p* = 0.220 at 1 month; −0.25, 95% CI = −0.40 to −0.90, *p* = 0.200 at 3 months) (Wu et al., 2017b). Alsaeid study of mild to moderate CTS, compared hyaluronidase with dexamethasone, showing significantly greater improvement in the hyaluronidase group at all follow-up time points (mean differences were: 0.70, 95%CI = -0.38 to -1.02, *p* < 0.050 at 1 week, −1.10, 95% CI = −0.65 to −1.55, *p* < 0.050 at 1 month; −1.30, 95%CI = −0.97 to −1.63, *p* = 0.030 at 3 months; −0.90, 95% CI = −0.42 to −1.38, *p* < 0.050 at 6 months) (Alsaeid, 2019). Güven et al. studied in mild to moderate CTS, compared PRP plus splinting with splinting alone, delta analysis showed significantly greater improvement in the PRP plus splinting group (*p* = 0.005) (Güven et al., 2019). Shen et al. studied moderate CTS, compared PRP with D5W, the results showed significantly greater improvement in the PRP group at six months (mean differences: −0.4, 95% CI = −0.45 to −1.25, *p* = 0.112) (Shen et al., 2019).

Sensory latency was measured in a study by Senna et al. There was significantly greater improvement in the PRP group than methylprednisolone group at three months (mean difference: −1.40, 95% CI = −1.11 to −1.69, *p* < 0.001) (Senna et al., 2019). Distal CMAP amplitude and SNAP amplitude was measured also in a study by Senna et al. However, there were no significant differences between the PRP and methylprednisolone groups (Senna et al., 2019).

A study by vanVeen et al. measured MNCV of ulnar nerve across the elbow and MNCV slowing across the elbow. However, there were no significant differences between the methylprednisolone and NSS groups (van Veen et al., 2015).

### Effect on Nerve Cross-Sectional Area (CSA)

All ten studies measured the CSA of the studied nerve (van Veen et al., 2015; Wu et al., 2017a; Wu et al., 2017b; Malahias et al., 2018; Roghani et al., 2018; Wu et al., 2018; Alsaeid, 2019; Güven et al., 2019; Senna et al., 2019; Shen et al., 2019). Two of the studies showed significant different improvements between groups in longer follow-up assessments (at 3 and 6 months), one was a study by Wu et al., 2017b and another was by Shen et al. (Wu et al., 2017b; Shen et al., 2019). A study by Wu et al., 2017b, comparing D5W with NSS as a control, showed significantly greater improvement in dextrose group than NSS group (mean difference: −1.83, 95% CI = −0.89 to −2.77, *p* < 0.001 at 3 months; −2.11, 95% CI = −1.11 to −3.09, *p* < 0.001 at 6 months) (Wu et al., 2017b). In a study by Shen et al., comparing PRP with D5W, showed significantly greater improvement in PRP group than dextrose group (mean difference: −2.9, 95% CI = −1.33 to −4.47, *p* < 0.001 at 3 months; −3.3, 95% CI = −1.73 to −4.87, *p* < 0.001, at 6 months) (Shen et al., 2019). A study by Wu et al. 2017(a), comparing PRP with splinting, showed significant improvement between groups at all follow up time points at 1, 3 and 6 months (mean difference: −2.15, 95% CI = −0.09 to −4.39, *p* < 0.001 at 1 month; −2.66, 95%CI = −0.45 to −4.87, *p* < 0.001 at 3 months; −3.08, 95%CI = −0.86 to −5.30, *p* < 0.001 at 6 months) (Wu et al., 2017a).

### Safety Outcomes

Adverse effects were reported in only one study on ulnar nerve entrapment at the elbow by vanVeen et al.; comparing methylprednisolone and NSS. Five patients reported a complication. One of the five patients received a placebo and reported pain at the site of injection (n = 25 in the placebo group, 4%). Four patients were treated with methylprednisolone, one reported swelling at the injection site, one had pain at the injection site, one had a swollen hand, and one had depigmentation at the injection site (n = 30 in methylprednisolone group, 13.3%) (van Veen et al., 2015). One study did not report adverse effects (Roghani et al., 2018). Seven studies reported no complications, nerve trauma, or serious adverse effects observed during the study (Wu et al., 2017a; Wu et al., 2017b; Malahias et al., 2018; Wu et al., 2018; Güven et al., 2019; Senna et al., 2019; Shen et al., 2019). One study reported no allergy to hyaluronidase (Alsaeid, 2019).

## Discussion

To the author’s knowledge, this study is the only systematic review selecting only ultrasound-guided hydrodissection articles. This systematic review retrieved ten eligible studies on ultrasound-guided hydrodissection for treatment of entrapment neuropathy with different injectates (van Veen et al., 2015; Wu et al., 2017a; Wu et al., 2017b; Malahias et al., 2018; Roghani et al., 2018; Wu et al., 2018; Alsaeid, 2019; Güven et al., 2019; Senna et al., 2019; Shen et al., 2019). The majority of studies were conducted in patients with mild to moderate carpal tunnel syndrome (CTS), the most common entrapment neuropathy (Wu et al., 2017a; Wu et al., 2017b; Malahias et al., 2018; Roghani et al., 2018; Wu et al., 2018; Alsaeid, 2019; Güven et al., 2019; Senna et al., 2019; Shen et al., 2019). All studies compared different interventions with different factors, none of the studies could be matched, therefore, a pairwise or network meta-analysis was infeasible. The authors selected studies using ultrasound-guided hydrodissection so that any clinical effect differences would unlikely result from needle misplacement, minimizing interference with result evaluation. Injectates used in the selected studies were normal saline, local anesthetics, corticosteroids, dextrose, platelet-rich plasma, and hyaluronidase. Each injectate offered different clinical effects of interest including pain, clinical symptoms, and function, physical performance, electrodiagnostic findings, and nerve cross-sectional area because of various mechanisms, both mechanical decompression effect and pharmacologic effects of the injectates. Each injectate mechanism was described in the following paragraphs.

Normal saline (NSS) or 0.9% sodium chloride (NaCl) is a crystalloid fluid with an osmolarity of 30.8 mOsmol/L and a pH range of 4.5–7. Within every 100 ml of 0.9% sodium chloride injection, there is an equal amount (154 mEq) of sodium and chloride ions (Baxter Corporation, 2016; Tonog and Lakhkar, 2020). For hydrodissection purposes, it can be used on its own or as a diluent for other injectates, for example, corticosteroids or local anesthetics, acting mainly as perineural space expander without intrinsic inflammatory reducing or nerve repairing effects (Chang et al., 2020). Of the 10 studies, three studies used NSS as a control injectate compared with methylprednisolone (van Veen et al., 2015), D5W (Wu et al., 2017b), and PRP (Malahias et al., 2018).

Local anesthetic (LAs) is the primary pain-reducing agent for the procedure, often serving as a combination agent with steroids (Chang et al., 2020). Local anesthetics share the same chemical composition (pharmacophore) of three structural domains: an aromatic group, a terminal amine group, and a hydrocarbon chain being ester or amide linkage connecting these two groups. Therefore, they are classified structurally as ester-linked LAs or amine-linked LAs (Tetzlaff, 2000; Page et al., 2006). From the included studies in this systematic review, the most commonly used agent for hydrodissection was lidocaine, ranging from 1–2% concentration with injected volume of 1–2 ml (van Veen et al., 2015; Wu et al., 2017b; Malahias et al., 2018; Roghani et al., 2018; Güven et al., 2019; Shen et al., 2019). Only one study used 3 ml of 0.5% bupivacaine (Alsaeid, 2019). Both agents belong to amide-linked LAs and the preparation was without vasopressors. LAs reduce pain directly by reversibly blocking voltage-gated sodium channels within an axon, especially the axons of afferent nociceptors, which are Aδ-fibers and C-fibers, these fibers play a major role in pain perception. Lidocaine has pKa lower than bupivacaine, 7.9 *vs* 8.1, respectively, this allows more rapid onset, moderate hydrophilicity allowing moderate potency and adequate duration of action of around 1–2 h. Because of higher pKa, bupivacaine provides slower onset and much longer duration of action and higher potency (Becker and Reed, 2006; Becker and Reed, 2012; Schulman and Strichartz., 2012). In addition to anesthetic properties, LAs may play an anti-inflammatory role as reported in a systematic review and may be considered as a single agent for hydrodissection when steroid is less preferred, for example, in elderly patients with diabetes mellitus (Caracas et al., 2009; Roghani et al., 2018).

Corticosteroids are a strong anti-inflammatory agent and provide pain relief mainly through anti-inflammatory mechanisms including inhibitory effects on cytokines, reducing inflammatory mediators such as leukotrienes, prostaglandins, and platelet-activating factors, preventing the recruitment and activation of several inflammatory cells including lymphocytes, eosinophils, basophils, and macrophages (Guyre et al., 1988; Barnes et al., 1993). Corticosteroids also reduce edema by reducing capillary permeability and blood flow, and also reduce granulation tissue formation (Schwiebert et al., 1996). Synthetic steroid preparations for local injection are available with varying anti-inflammatory potencies, glucocorticoid effect, mineralocorticoid activities, solubility, and duration of actions. Commonly used injectable steroids, such as triamcinolone, methylprednisolone, and dexamethasone are derivatives of prednisolone. They are compounds with an-OH (hydroxyl) group, having intrinsic glucocorticoid property, and are ready to act without prior conversion in the liver (Garg and Adler, 2012). The first two preparations are in microcrystalline suspension form with extensive particle aggregation while dexamethasone preparation is in clear solution form. The particulate form potentially gives a longer duration of action than the non-particulate form as the particles were slowly released (MacMahon et al., 2009). Of the ten studies, five used corticosteroids; one used dexamethasone (Alsaeid, 2019), two used triamcinolone (Roghani et al., 2018; Wu et al., 2018), and two used methylprednisolone (van Veen et al., 2015; Senna et al., 2019) with injected volume ranging from 1–2 ml. From the described mechanism, corticosteroids provide a clinical effect of pain reduction, improving symptoms, decreased CSA due to edema reduction, allowing more space around the nerve, enable the electrophysiologic findings to improve.

Five percent dextrose in water or D5W is an isotonic solution of dextrose in a form of d-glucose, containing 278 mmol/L of dextrose. How D5W relieves neuropathic pain in the perineural injection is still rather unclear. A hypothesis has been proposed that D5W relieves pain through a sensorineural mechanism by downregulating the transient receptor potential vanilloid receptor 1 (TRPV-1) which is usually upregulated in cases of chronic neuropathic pain (Malek et al., 2015; Reeves and Rabago, 2020). This hypothesis on the mechanism of pain reduction has been made from a pilot study using mannitol to reduce capsaicin-induced pain (Bertrand et al., 2015; Reeves et al., 2016). Another mechanism is by decreasing C-fibers activation by reversing hypoglycemic status which induces excessive C-fibers activation (MacIver and Tanelian, 1992). Even though there are studies that consistently report clinical benefits compared with injection control, evidence of nervous tissue proliferation remains unclear (Reeves and Rabago, 2020). Dextrose predominately provides pain reduction, and also improving symptoms, function, electrophysiologic findings, and CSA reduction. Of the ten studies, three studies used D5W for injectates, D5W is the intervention injectate of interest in two studies, one comparing with NSS control and one comparing with triamcinolone (Wu et al., 2017b; Wu et al., 2018), another study D5W was used as a comparative injectate against PRP (Shen et al., 2019) with injected volume range from 3 to 5 ml.

Platelet-rich plasma (PRP) is a portion of the plasma fraction of autologous blood with a platelet concentration above the baseline (before centrifugation). Once activated, secretory granules release many mediators important in homeostatic, growth factors, and cytokines affecting inflammation, angiogenesis, facilitating the natural healing process and promote regeneration in many tissue types (Andia and Abate, 2013; Alves and Grimalt, 2018). Growth factors important in promoting axonal regrowth and angiogenesis include nerve growth factor (NGF), brain-derived neurotrophic factor (BDNF), and transforming growth factor (TGF-β), vascular endothelial growth factor (VEGF), and insulin-like growth factor-1 (IGF-1) (Borselli et al., 2010; Kim et al., 2014). The PRP fraction may contain a supraphysiologic concentration of platelets ranging from two to five times the baseline concentration (Le et al., 2018). Due to different preparation protocols, yielded PRP component; platelet concentration, presence or absence of leukocytes and erythrocytes, and also the timing of activation, tends to vary from study to study (Lansdown and Fortier, 2017). By promoting axonal regrowth, PRP not only reduces pain but also restores the nerve’s function and preserves the properties of the target muscles (Frostick et al., 1998; Kuffler, 2013). Because of PRP’s regenerating mechanism, PRP provides broad clinical effect from pain reduction, improving symptoms, function, electrophysiologic findings as well as CSA reduction. Of the ten studies, two studies compared PRP with conservative measure, one compared PRP alone with splinting and another compared PRP plus splinting with splinting alone (Wu et al., 2017a; Güven et al., 2019). One study compared PRP with normal saline (Malahias et al., 2018), two studies compared PRP with another injectate being methylprednisolone and D5W (Senna et al., 2019; Shen et al., 2019). Injected PRP volume range from 2 to 3 ml. Only one study gave specific details of the PRP component describing 3 ml of injected PRP with a platelet concentration of 2.7 ± 0.4 times, leukocytes count 1.2 ± 0.4 (Wu et al., 2017a).

Hyaluronidase is a mucolytic enzyme derived from mammalian tissue or synthesized *in vitro* in pure form (rHuPH20) using recombinant technology. Hyaluronidase lowers the viscosity of hyaluronan, a constituent of the extracellular matrix, thereby increasing tissue permeability (Dunn et al., 2010). For hydrodissection purposes, it is used as an adhesiolysis agent to release the entrapped nerve. One study compared hyaluronidase (300 IU) with dexamethasone as an adjuvant to 0.5% bupivacaine, the clinical effect it provided included symptoms, electrophysiologic findings, and CSA improvement (Alsaeid, 2019).

From the selected studies, pain (VAS) reduction was significantly achieved greater than NSS control or splitting into studies using D5W and PRP (Wu et al., 2017a; Wu et al., 2017b). When comparing one injectate to another, one study showed greater VAS reduction in intervention injectate (D5W) comparing to triamcinolone (Wu et al., 2018), another study comparing PRP to methylprednisolone showed lower average VAS in the PRP group than methylprednisolone group at the three-month follow up (Senna et al., 2019). For clinical symptoms, function, and physical performance, the improvement was significantly greater than NSS control or splitting into studies using D5W and PRP (Wu et al., 2017a; Wu et al., 2017b; Malahias et al., 2018; Güven et al., 2019). When comparing one injectate to another, D5W, PRP, and hyaluronidase gave greater improvement than their steroids counterparts (Wu et al., 2018; Alsaeid, 2019; Senna et al., 2019). Regarding main electrodiagnostic parameters (SNCV and DML) findings, D5W and hyaluronidase resulted in superior outcomes comparing to NSS and dexamethasone, respectively (Wu et al., 2017b; Alsaeid, 2019). PRP plus splinting also significantly improved main electrodiagnostic parameters (Güven et al., 2019). Another PRP study evaluated sensory latency and PRP showed superior outcomes compared to dextrose (Shen et al., 2019). All studies measured studied nerve cross-sectional area, the greater reduction was observed using D5W with NSS control, and PRP with splinting control (Wu et al., 2017a; Wu et al., 2017b). One study showed that PRP also achieved greater CSA reduction than D5W (Shen et al., 2019). Different doses of corticosteroids did not result in significant differences between doses in any outcomes (Roghani et al., 2018). From the main findings, D5W gave consistently superior effects comparing to NSS control or triamcinolone across all outcomes measured with the greatest magnitude of difference in later follow-up months (3,4 or 6 months) (Wu et al., 2017b; Wu et al., 2018). PRP demonstrated superior pain, clinical symptoms, and CSA reduction when comparing to NSS or splinting (Wu et al., 2017a; Malahias et al., 2018). PRP plus splinting resulted in greater electrodiagnostic parameters improvement than splinting alone (Güven et al., 2019). Therefore, D5W and PRP could be considered the preferred injectates for mild to moderate CTS. This finding also corresponds to the recent meta-analysis investigating regenerative injections for CTS (Lin et al., 2020). It is noticeable that, in a study comparing the two (D5W vs PRP), both gave significant improvement after hydrodissection, significantly greater improvement parameters in the PRP group consisted of BCTQf, DML, and CSA (Shen et al., 2019). This is quite expected as both were effective, showing many significant outcome improvements comparing to NSS or splint control. Of note, is the recent injectate, hyaluronidase, giving superior effects comparing to dexamethasone in clinical symptoms and electrodiagnostic findings. Considering adverse events, the only study reported adverse event was ulnar nerve study using corticosteorids, the events were common side effects from local steroids injection including pain, swelling and depigmentation at the injection site (vanVeen et al., 2015). The other eight CTS studies reported no adverse events. Different anatomy of injected sites might explain the situation, as the tissue covering ulnar nerve at the elbow region is very thin and without structurally containing boundaries, the injectate may infiltrate after injection up to the subcutaneous layer, even with ultrasound guidance, unlike the median nerve which is located inside the carpal tunnel. Even though no studies report severe allergic reaction or systemic toxicity of injectates, there is still a potential for severe allergic reaction when injecting with local anesthetics, corticosteroids and hyaluronidase as the drug vehicles or preservatives in the preparation may provoke severe allergic reactions in some patients (MacMahon et al., 2009; Becker and Reed, 2012).

The most investigated injectate among nine CTS studies was PRP, being the intervention injectate in five studies (Wu et al., 2017a; Malahias et al., 2018; Güven et al., 2019; Senna et al., 2019; Shen et al., 2019), followed by dextrose, in two studies (Wu et al., 2017b; Wu et al., 2018). This has shown the trend toward the need for injectates with regenerative effects, expecting longer and more permanent recovery. As it is well-established now that corticosteroid injections in CTS provide good but short-term clinical symptoms relief. Even surgical treatment may not always restore the nerve function (Huisstede et al., 2010). One injectate that has just recently been seen in entrapment hydrodissection publications is hyaluronidase, primarily used in the ophthalmology field or for lysis of epidural adhesion, this was included in one of the selected studies (Dunn et al., 2010; Alsaeid, 2019). The only corticosteroid study in CTS was by Roghani et al., studying different doses of steroids compared with local anesthetics, still another pharmacologic agent, as a control group. This study particularly aimed at finding the optimal corticosteroid dose for use in elderly patients, different from other corticosteroid studies (Roghani et al., 2018). Interesting findings from the study was the control group (local anesthetics alone) experienced significant pain reduction, improved symptoms, and reduced CSA like the steroids group. The authors proposed that this may result from the potential anti-inflammatory effect of local anesthetics (Roghani et al., 2018). The only study investigating the effect of corticosteroids compared with a normal saline control was by vanVeen et al. As ulnar nerve entrapment is less common than CTS, less publications with much less controlled-trials publications exist. Corticosteroids remain the primary investigated or reported agent for ulnar nerve entrapment, therefore, possibilities exist for investigating other types of injectates. The challenges when evaluating PRP studies remained the undetermined dosage of platelets in PRP as many studies did not provide a full description. For studies using NSS as the control group, there was also a noticeable improvement in the group, implying the effectiveness of hydrodissection partly did come from a purely mechanical decompression. This effect was demonstrated in a randomized controlled trial study comparing ultrasound-guided hydrodissection with NSS and subcutaneous injection with NSS (Wu et al., 2019). Considering the potential local and transient blood sugar elevation side effects of steroid injections, especially in the elderly or patients with elevated blood sugar, D5W or PRP might be a more preferable option for these groups.

There are several limitations in this systematic review, first, all ten studies compared different interventions and comparisons, none could be combined for further analysis. Second, of ten studies, three were from the same investigator's group, this might limit the generalization of results as the study population was limited. Third, the follow-up interval was rather diverse with a maximum follow-up time at six months, which might be insufficient for evaluating regenerative effects. Fourth, the varying injected volume among the studies might also vary the clinical outcome as larger volume tends to provide greater mechanical decompression.

To further enhance knowledge of ultrasound-guided hydrodissection procedure, more studies on different nerves and locations are encouraged as well as in varied population groups to promote generalizability. Also, for PRP and D5W studies of longer duration than six months should be pursued. For future PRP studies, a full PRP preparation protocol together with detailed PRP components should be explained thoroughly as the information will be very helpful when comparing studies.

## Conclusion

In summary, this systematic review shown the effectiveness and safety of ultrasound-guided hydrodissection injectates ranging from NSS, D5W, local anesthetics, corticosteroids, PRP, and hyaluronidase. All injectates can provide a clinical effect on their own. In comparative cases, D5W and PRP demonstrated a consistent superior clinical effect against the comparative agent or other conservative measures. With ultrasound-guidance, no serious adverse events occurred, except local side effects after corticosteroid injections.

## Data Availability

The original contributions presented in the study are included in the article/Supplementary Material, further inquiries can be directed to the corresponding author.

## Abbreviations:

2PD, 2-point discrimination; BCTQ, boston carpal tunnel questionnaire; BCTQs, boston carpal tunnel questionnaire symptom severity scale; BCTQf, boston carpal tunnel questionnaire functional status scale; BDNF, brain-derived neurotrophic factor; CGRP, calcitonin gene related peptide; CMAP, compound muscle action potential; CSA, cross-sectional area; CTS, carpal tunnel syndrome; CuTS, cubital tunnel syndrome; D5W, 5% dextrose in water; DML, distal motor latency; EDS, electrodiagnostic study; IGF-1, insulin-like growth factor-1; IL, interleukin; LAs, local anesthetics; MeSH, medical subject headings; MNCS, motor nerve conduction study; NaCl, sodium chloride; NGF, nerve growth factor; NSS, normal saline; OH, hydroxyl; PRISMA, preferred reporting items for systematic reviews and meta-analyses; PRP, platelet-rich plasma; RoB, risk-of-bias; Q-DASH, quick disabilities of arm shoulder and hand questionnaire; SNAP, Sensory nerve action potential.
